# Cannabis use is not associated with increased balance disturbances in HIV-infected individuals

**DOI:** 10.1186/s42238-021-00059-z

**Published:** 2021-02-03

**Authors:** Patrick Kiendrebeogo, David Grelotti, Mariana Cherner, Raeanne C. Moore, Bin Tang, Ronald J. Ellis

**Affiliations:** 1 Interdisciplinary Research Fellowship, University of California San Diego, San Diego, USA; 2 University of California San Diego, San Diego, USA

**Keywords:** HIV infection, Cannabis, Balance disturbances, Falls

## Abstract

**Background:**

The association between long-term cannabis use and balance disturbances has not been investigated in people living with HIV (PWH). We hypothesized that long-term cannabis use in PWH might be associated with more deleterious effects on balance than in HIV seronegative individuals due to potential neurotoxic interactions between HIV and cannabis.

**Methods:**

Three thousand six-hundred and forty-eight participants with and without HIV completed an interviewer-administered timeline follow-back assessment to assess lifetime days and quantity of cannabis use and other cannabis use characteristics. A structured clinical interview was used to collect any history of balance disturbance. Comparisons between HIV+ vs the HIV− groups and moderate-severe vs. no or minimal imbalance in participant characteristics (demographics, cannabis use, medication currently used, and neurological disease) were performed using Student *t* tests for continuous variables and Fisher’s exact test for binary and categorical variables. Multivariate logistic regression was applied to determine the interaction effect of total quantity of cannabis use with HIV status on balance disturbance. Age, gender, cDSPN symptoms, gait ataxia, opioid medications, and sedatives were included as covariates in the adjusted model after variable selection. The effect sizes are presented as Cohen’s *d* or odds ratios.

**Results:**

On average, participants were 45.4 years old (SD = 11 years), primarily male (77.7%), and non-Hispanic white (48.1%). A majority of participants were HIV+ (79.1%). Four hundred thirty (11.9%) of the participants reported balance disturbances within the past 10 years. PWH were more likely to have balance disturbances than demographically matched HIV-uninfected participants (odds ratio [OR] 2.66, 95% CI 1.91–3.7). Participants with moderate-severe balance disturbances did not differ from those with no or minimal imbalance in the proportion who had ever used cannabis (73.8% vs. 74.4%; *p =* 0.8) (OR 1.03, 95% CI 0.80–1.32) neither did they have a higher total amount of cannabis use (4871 vs. 4648; *p* = 0.3) (Cohen’s *d* 0.11, 95% CI 0.01–0.14). In the HIV− population, those with balance disturbances reported more total amount of cannabis use as compared to those with normal balance (11316 vs 4154; *p* = 0.007). In the HIV+ population on the other hand, there was no significant association (4379 vs 4773; *p* = 0.6).

**Conclusions:**

We found unexpectedly that while long-term cannabis use in HIV− individuals was associated with more severe balance disturbances, there were no associations in HIV+ individuals. This suggests that cannabis use in HIV is safe with respect to balance disturbances. Given that HIV is related to persistent inflammation despite virologic suppression on antiretroviral therapy, future mechanistic studies are needed to determine whether HIV-associated inflammation contributes to the higher prevalence of balance disturbance in HIV+ individuals and whether cannabinoids have anti-inflammatory effects that mitigate HIV-associated balance disturbance.

## Introduction

According to the Center for Disease Control data (Ellis et al. 2011), falls are the leading cause of fatal and nonfatal injuries among adults aged ≥ 65 years (older adults). For older adults, falls and associated injuries threaten their health, independence, and quality of life. More than a third of people aged 65 and older living independently fall each year (Tromp et al. 2001), representing a major public health problem. Aging HIV+ individuals have an increased prevalence of many fall-related risk factors, and a study has previously shown that the fall rate among middle-aged (45–65 years) HIV+ individuals on effective antiretroviral therapy (ART) mirrors that of uninfected adults aged 65 or older (Nakatsukasa et al. 2014). In addition to their high risk of falls, HIV+ individuals may be at a greater risk of sustaining an injurious fall or fracture due to underlying low bone density, low body weight, peripheral neuropathy, neurocognitive impairment, and frailty (Erlandson et al. 2017).

Cannabis is used recreationally as well as for different medical indications among HIV+ individuals (e.g., improvement of appetite, pain, sleep) and studies have shown an improvement of neuropathic pain in HIV+ individuals using cannabis (Ellis et al. 2011). According to a National Academies of Sciences, Engineering, and Medicine 2017 report, there is conclusive or substantial evidence for the use of cannabis for the treatment of chronic pain (Hea 2017). Cannabis use is also legal for recreational purposes in many states in the USA, which has likely increased its use in the general population. The active components in cannabis are known as cannabinoids, and the main cannabinoids are tetrahydrocannabinol (THC) and cannabidiol (CBD). Cannabinoid receptors (CB1 and CB2) are expressed in the brain and are involved in its health and disease; CB1 receptors are found in the brain region that mediate the control of balance (basal ganglia, cerebellum, neocortex) and CB2 receptors are found in immune cells in the brain, playing a role in neuroinflammation (microglia) (Kendall and Yudowski 2017). THC and CBD interact with these receptors, thereby influencing balance and neuroinflammation. THC is the primary psychoactive component in cannabis, and it is associated with variable degrees of drowsiness (C. Database and S. Reviews 2017), dizziness, and sedation (La and Ce 2011), which alone or together could contribute to imbalance and consequently to falls during acute intoxication. By way of contrast, CBD is the major non-psychoactive component of cannabis and has been shown to be anti-inflammatory in models (Costa et al. 2004; Juknat et al. 2016; Petrosino et al. 2018).

Despite the potential health benefits of cannabis use for HIV infection, the relationship between long-term cannabis use and balance disturbances remains unknown. In this study, we compared the prevalence of balance disturbances among HIV+ and HIV− cannabis users, controlling for relevant covariates. We hypothesized that long-term cannabis use in HIV+ individuals might be associated with more severe balance disturbances than in HIV− individuals due to potential neurotoxic interactions between HIV infection and cannabis.

## Methods

### Participants and design

The study comprised 3664 ambulatory HIV+ and HIV− individuals enrolled in multiple NIH-funded research studies at the University of California, San Diego HIV Neurobehavioral Research Program (HNRP). Participants were enrolled between September 2003 and June 2017, and the most recent evaluation was used for each participant. At the time of enrollment, all participants provided written, informed consent. Secondary data analysis was performed. Inclusion criteria for this analysis included completion of a structured clinical interview which provided details regarding the occurrence of cannabis use and balance disturbances and completion of a neurological examination. The clinical interview and the physical examination were performed on all participants. Exclusion criteria included blindness, being a wheelchair user and experiencing falls as a consequence of sustaining a violent blow, loss of consciousness or sudden onset of paralysis as in stroke or epilepsy. We excluded individuals with other neurologic conditions such as motor neuron disease, Parkinson disease, and multiple sclerosis. Individuals with stroke were excluded only if they had persistent neurological deficits after their stroke. Recognizing that peripheral neuropathy and vestibular disease are common in HIV+ individuals, we did not exclude these conditions. Additionally, urine samples were collected at screening and participants with a positive toxicology report (except for cannabis) were excluded.

### Clinical evaluation

#### Cannabis use

Cannabis and other substance use data were collected using the interviewer-administered timeline follow-back assessment (TLFB), a gold-standard measure for retrospectively assessing detailed alcohol and drug use characteristics. The TLFB uses a calendar method to evaluate daily patterns and frequency of substance use over a specified period. It has high retest reliability, convergent and discriminant validity with other measures, agreement with collateral informants’ reports of participants’ substance use, and agreement with urine toxicology assays (Fals-Stewart et al. 2000). Cannabis recency was assessed as self-reported 12 h since last cannabis use. Other cannabis variables assessed self-reported frequency, density, cumulative dose, and total years of cannabis use. For the present analysis, we used the total quantity (grams) of cannabis use as the predictor variable. The main study aim was to assess interactions between HIV infection and long-term cannabis use on balance disturbance; therefore, total quantity of cannabis use as predictor provides an estimate of potential cumulative toxicity. We used the Composite International Diagnostic Interview version 2.1. (CIDI) to reliably assess substance use disorder. 

#### Balance disturbance

A structured clinical interview was administered to participants by trained interviewers to collect any history of balance disturbance. Inter-examiner reliability was ensured through systematic training. Participants were asked about balance problems in the past few days up to the previous 10 years. Balance disturbances were self-reported and classified according to their severity into the following categories: (1) normal; (2) occasionally unsteady, and no falls; (3) frequently unsteady, some near-falls, and rare falls; and (4) must use a cane, walker, or other prop. We recoded balance disturbances into no or minimal balance disturbances (normal and occasionally unsteady, no falls) and moderate-severe balance disturbances (frequently unsteady, some near-falls, rare falls, and must use a cane, walker, or other prop). This method has been previously used in a study of the influence of distal sensory polyneuropathy on balance disturbances in HIV+ individuals (Sakabumi et al. 2019). The presence or absence of ataxia was assessed during the gait examination (the participants were asked to walk as briskly as could be done safely for 10–20 feet, turn, and return to the starting point).

#### HIV characteristics and covariates

We collected data on HIV disease characteristics including current and nadir CD4 count, plasma viral load < 50 copies/mL (undetectable viral load), duration of HIV infection, historical AIDS status, and current use of ART. We asked about the use of medications commonly associated with balance problems: antihypertensives, sedatives, and opioids. We also collected data on age, gender, race/ethnicity, and education. History of long-term alcohol abuse and diabetes were also reported. Height and weight were measured in order to calculate the body mass index (BMI). Chronic distal sensory polyneuropathy (cDSPN) was diagnosed based on the presence of any of the following abnormal findings in a distal (feet and toes more than calves and thighs), symmetrical distribution during physical examination: reduced sharp sensation, vibration sense, or reflexes.

### Statistical analysis

Comparisons between HIV+ vs. HIV− groups and moderate-severe vs. no or minimal imbalance in participant characteristics (demographics, cannabis use, medication currently used, and neurological disease) were performed using Student *t* tests for continuous variables and Fisher’s exact test for binary and categorical variables. Using similar methods, HIV disease characteristics were compared in HIV+ individuals with moderate-severe vs. no or minimal imbalances. Prior to statistical analyses, current and nadir CD4 counts (cells/μl) were square root transformed to better fit a normal distribution. Multivariate logistic regression was applied to determine the interaction effect of total quantity (grams) of cannabis use with HIV status on balance disturbance. Age, gender, cDSPN symptoms, gait ataxia, opioid medications, and sedatives were included as covariates in the adjusted model after variable selection. The effect sizes are presented as Cohen’s *d* or odds ratios; Cohen’s *d* was calculated by dividing the difference of means by the root-mean-standard-error (RMSE) and the odds ratios were used to quantify effect sizes for nominal variables. Statistical analyses were completed with JMP Pro 14. Alpha was set at 0.05.

## Results

Descriptive statistics are provided in Table 1. On average, participants were 45.4 years old (SD = 11 years), primarily male (77.7%), and non-Hispanic white (48.1%). A majority of participants were HIV+ individuals (79.1%). 75.1% of the participants reported normal balance, 13.0% were occasionally unsteady, 8.2% were frequently unsteady, and 3.7% had to use a cane/walker. Based on our categorization, 430 (11.9%) participants reported balance disturbances within the past 10 years (Table 2). HIV+ individuals were more likely to have balance disturbances than HIV− individuals (odds ratio [OR] 2.66, 95% CI 1.91–3.7).

**Table 1:** Participant characteristics stratified by HIV status

Variables	HIV+ individuals (*n* = 2887)	HIV− individuals (*n* = 761)	Effect size (95% CI)	*p* value
Demographics				
Age (years), mean (SD)	45.6 (11)	44.9 (14)	0.05 [− 0.02; 0.13]^a^	0.9
Education, mean (SD)	12.9 (3)	13.6 (4)	− 0.19 [− 0.27; − 0.11]^a^	< .0001
Male, *n* (%)	2333 (80.8%)	501 (65.8%)	2.18 [1.83; 2.61]^b^	< .0001
Ethnicity, *n* (%)				
Hispanic	503 (17.4)	147 (19.3%)		< .0001
Non-Hispanic white	1328 (46)	430 (56.5%)		
African American	958 (33.2)	136 (17.9%)		
Asian	25 (0.9)	14 (1.9)		
Other	73 (2.5)	34 (4.5)		
BMI (kg/m^2^), mean (SD)	26.6 (10)	28.3 (6)	− 0.17 [− 0.29; − 0.09]^a^	< .0001
Cannabis use, *n* (%)				
Cannabis ever used, *n* (%)	1821 (74.9)	408 (72.6)	0.88 [0.72; 1.09]^b^	0.2
Lifetime years cannabis use, mean (SD)	6.9 (8.4)	7.2 (9.5)	− 0.03 [− 0.14; 0.07]^a^	0.5
Lifetime quantity cannabis (grams), mean (SD)	4724 (12775)	4515 (11627)	0.01 [− 0.09; 0.12]^a^	0.6
Medication currently used, *n* (%)				
Opioids, *n* (%)	480 (16.6)	53 (6.9)	2.66 [1.98; 3.58]^b^	<.0001
Sedatives, *n* (%)	365 (12.6)	39 (5.1)	2.75 [1.95; 3.87]^b^	<.0001
Antihypertensives, *n* (%)	223 (7.7)	65 (8.5)	0.9 [0.67; 1.21]^b^	0.5
Neurological disease, *n* (%)				
cDSPN signs, *n* (%)	1574 (54.5)	190 (24.9)	3.60 [3.00; 4.31]^b^	<.0001
Gait ataxia, *n* (%)	130 (4.5)	20 (2.6)	1.74 [1.08; 2.81]^b^	0.02

Effect size presented as ^a^Cohen’s *d* or ^b^odds ratios

*CI* confidence interval, *BMI* body mass index, *cDSPN* chronic distal sensory polyneuropathy

**Table 2:** Participant characteristics stratified by severity of balance disturbance

Variables	Moderate-severe Imbalance (*n* = 430)	No or minimal Imbalances (*n* = 3191)	Effect size (95% CI)	*p* value
Demographics				
Age (years), mean (SD)	51.7 (10)	44.8 (11)	0.62 [0.52; 0.72]^a^	< .0001
Education, mean (SD)	12.9 (3)	13.0 (3)	− 0.03 [− 0.13; 0.06]^a^	0.756
Male, *n* (%)	306 (71.2)	2504 (78.5)	0.67 [0.54; 0.84]^b^	< .0006
Ethnicity, *n* (%)				
Hispanic	54 (12.6)	590 (18.5)		0.04
Non-Latino white	216 (50.2)	1524 (47.8)		
African American	144 (33.5)	949 (29.7)		
Asian	4 (0.9)	33 (1.0)		
Other	12 (2.8)	95 (3.0)		
BMI (kg/m^2^), mean (SD)	27.9 (7)	26.9 (9)	0.10 [0.00; 0.20]^a^	0.006
Cannabis use, *n* (%)				
Urine toxicology positive for THC	63 (14.9)	451 (14.27)	1.09 [0.82; 1.46]^b^	0.5
Cannabis ever used, *n* (%)	262 (73.8)	1964 (74.4)	1.03 [0.80; 1.32]^b^	0.8
Lifetime years Cannabis use, mean (SD)	9.7 (7.8)	8.4 (6.8)	0.12 [− 0.00; 0.25]^a^	0.07
Lifetime Quantity Cannabis (grams), mean (SD)	4871 (11573)	4648 (12705)	0.11 [0.01; 0.14]^a^	0.3
HIV serostatus, *n* (%)				
HIV+ individuals	384 (13.5)	2462 (86.5)		
HIV− individuals	42 (5.5)	717 (94.6)		
Neurological disease, *n* (%)				
cDSPN, *n* (%)	303(70.5)	1469 (46.0)	2.79 [2.24; 3.47]^b^	< .0001
gait ataxia, *n* (%)	54 (12.5)	96 (3.0)	0.2 [0.15; 0.30]^b^	< .0001
Medication currently used, *n* (%)				
Opioids, *n* (%)	132 (30.7)	401 (12.6)	3.08 [2.44; 3.87]^b^	< .0001
Sedatives, *n* (%)	77 (17.9)	327 (10.2)	1.99 [1.51; 2.62]^b^	< .0001
Antihypertensives, *n* (%)	52 (12.1)	235 (7.4)	1.77 [1.29; 2.44]^b^	< .0001

Effect size presented as ^a^Cohen’s *d* or ^b^odds ratios

*CI* confidence interval, *BMI* body mass index, *cDSPN* chronic distal sensory polyneuropathy

HIV+ individuals had more severe balance disturbances than HIV− individuals: for occasionally unsteady, HIV+ individuals 14.7% vs HIV− individuals 6.85%; for frequently unsteady, 9.3% vs 4.0%; and for must use cane or walker, 4.18% vs 1.58% (Cochran Armitage Trend Test *z* = − 8.18, *p =* 2.88e− 16). To simplify additional analyses and comparisons of effect sizes using odds ratios, for further analyses we used the groups normal and occasionally unsteady as compared to frequently unsteady and must use cane or walker. Table 2 shows that those with more severe balance disturbances were more likely to be older, female, and slightly heavier; have cDSPN; have gait ataxia; and use medications with potential to cause imbalance (opiates, sedatives, antihypertensives). Participants who had more severe balance disturbances were more likely to use antidepressant (17.4% vs 9.30%; *p* < 0.0001). A history of diabetes was also associated with a higher likelihood of balance disturbance. There was no significant effect of long-term alcohol abuse on balance disturbances in our population. Participants with moderate-severe balance disturbances did not differ from those with no or minimal imbalance in the proportion who had ever used cannabis (73.8% vs. 74.4%; *p =* 0.8) (OR 1.03, 95% CI 0.80–1.32) neither did they have a higher total quantity (grams) of cannabis use (4871 vs. 4648; *p* = 0.3) (Cohen’s *d* 0.11, 95% CI 0.01–0.14).

Among HIV+ individuals, factors related to HIV including positive AIDS status, lower nadir CD4, current ART treatment, and a longer duration of HIV infection were statistically significantly associated with a higher likelihood of balance disturbances (Table 3). Participants on efavirenz (EFV) had a reduced odds of balance problems compared to those on other ARVs (OR 0.652 [0.4840.880]), but an increased risk compared to those off ARVs (OR 1.41 [1.00, 1.97]). The proportion with an undetectable viral load in the entire HIV+ population was 58.6% (58.7% with moderate-severe imbalance vs 58.6% with no or minimal imbalance).

**Table 3:** HIV disease and treatment characteristics of persons living with HIV according to balance disturbances

Variables	HIV+ individuals with moderate-severe imbalance (*n* = 384)	HIV+ individuals with no or minimal imbalance (*n* = 2462)	Effect size (95% CI)	*p* value
AIDS, *n* (%)	286 (74.5%)	1510 (61.3%)	1.83 [1.44; 2.34] ^b^	<.0001
Current CD4(cells/μL), median [IQR]	477 [279, 696]	469 [301, 685]	0.00 [− 0.10; 0.11] ^a^	0.9
CD4 nadir(cells/μL), median [IQR]	107 [20, 239]	171 [40, 299]	− 0.19 [− 0.3; − 0.09] ^a^	<.0002
Undetectable viral load, *n* (%)	203 (58.7%)	1355 (58.6%)	0.99 [0.78; 1.25] ^b^	0.902
Currently on ART, *n* (%)	309 (80.7%)	1908 (77.6%)	1.65 [1.08; 2.54] ^b^	<.02
Duration of HIV infection (years), mean (SD)	14.0 (13)	11.6 (11)	0.23 [0.12; 0.34] ^a^	<.0005

Effect size presented as ^a^Cohen’s *d* or ^b^odds

*CI* confidence interval, *AIDS* acquired immune deficiency syndrome, *HIV* human immunodeficiency virus, *ART* antiretroviral therapy

In the HIV− population, those with balance disturbances reported more total amount of cannabis use as compare to those with normal balance (11316 vs 4154; *p* = 0.007). In the HIV+ population on the other hand, there was no significant association (4379 vs 4773; *p* = 0.6).

**Fig. 1:**
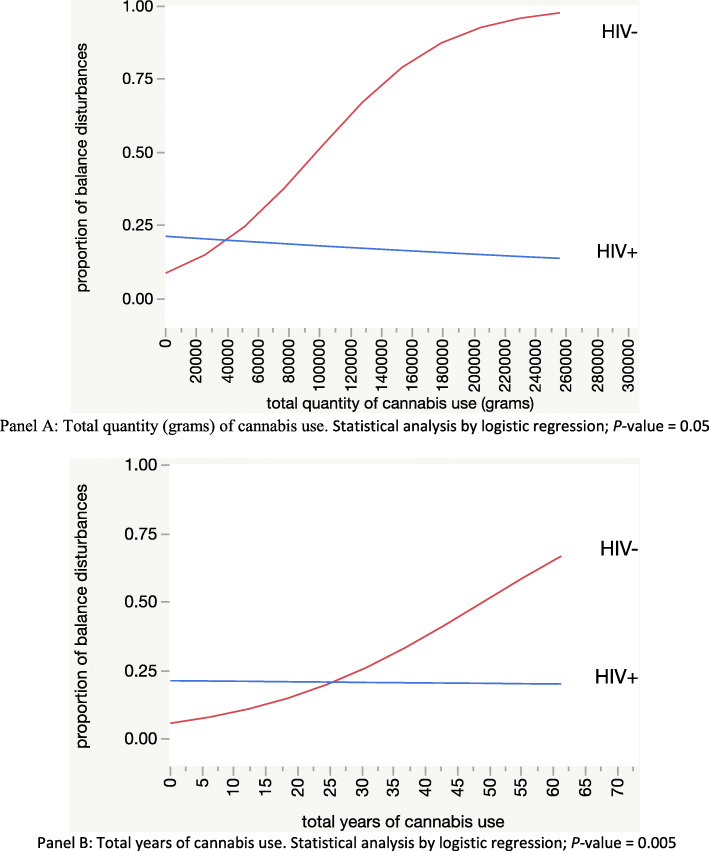
Interaction of total quantity (**a**) and years (**b**) of cannabis use with HIV status as regards balance disturbance (self-report using TLFB method for cannabis variable; structured interview for balance disturbance)

Controlling for age, gender, cDSPN symptoms, gait ataxia, opioid medication, and sedatives, we evaluated the relationship between self-reported balance disturbances and cannabis use by HIV status. We observed a statistically significant interaction between HIV status and total quantity (grams) of cannabis use as regards balance disturbances such as while total quantity of cannabis use was associated with more severe balance disturbances in HIV− individuals, it was unrelated to balance disturbances in HIV+ individuals (Fig. 1). In a sensitivity analysis, we found similar results after excluding participants with more severe balance disturbances (must use cane/walker). Additionally, we evaluated very recent self-reported cannabis use and found the same interaction (*p =* 0.0494) such that among HIV− individuals, those who used in the past 12 h were more likely to have balance disturbances than those not using in the past 12 h (30.0% vs 3.4%; Fisher’s exact *p =* 0.0001); for HIV+ individuals, there was no significant difference according to recency of cannabis use (17.4% vs 11.1%, Fisher’s exact *p =* 0.18).

## Discussion

Contrary to our hypothesis, this study provides evidence that more extensive, long-term cannabis use among HIV+ individuals is not associated with a higher likelihood of balance disturbances. While we did not find any research study in the literature to compare with our findings, one prior report found that the occurrence of balance disturbances was associated with a 13-fold higher odds of recurrent falls among HIV+ individuals (Nakatsukasa et al. 2014). It is not clear why the more frequent use of cannabis was not associated with a higher likelihood of balance disturbances in the HIV+ group, but a plausible explanation is that any deleterious effects of cannabis are counteracted by its effect of reducing inflammation (Juknat et al. 2012; Manuzak et al. 2018). Yet, the difference between HIV+ and HIV− individuals in cannabis-associated balance disturbances as both acute effect and chronic effect suggest that neuroinflammatory differences alone may not explain these results. In the brain, CB2 receptor expression is associated with inflammation and it is primarily localized to microglia (resident macrophages of the brain). This selective localization together with the modulatory effect of the CB2 receptor on microglia function (Kendall and Yudowski 2017) is particularly relevant since microglial cells have a significant role in neuroinflammation in HIV infection. In fact, HIV-infected monocytes not only infect brain resident cells (microglia) upon migration into the CNS but also produce proinflammatory cytokines, which in turn, further activate microglia. These activated microglia, along with perivascular macrophages, are the main contributors to neuroinflammation in HIV infection, resulting in neuronal dysfunction and death (Hong and Banks 2015).

In contrast, more prolonged chronic cannabis use was related to more severe imbalance among the HIV− individuals. One potential mechanism for this is adverse effects on the cerebellum and basal ganglia, both of which express high levels of CB1 receptors. Prior research (Bolbecker et al., 2018) found that chronic cannabis use in HIV− individuals was associated with increased postural sway in individuals who were not acutely intoxicated. Our results are similar to those of Bidwell et al. (2020) who found that balance function was impaired after immediate cannabis use and different from those of Pearson-Dennett et al. (2017) who found that the effect of long-term cannabis use was associated with long-lasting changes in open-chain elements of walking gait, but the magnitude of change was not clinically detectable.

Those studies assessed balance impairment after immediate cannabis use in small samples. In contrast, our study focused on prolonged use of cannabis and had more power due to the large population size. In Pearson-Dennett et al.’s (2017) study the average population age was between 24 and 25 years; however, in our study the average population was 45.4 years and any subtle walking gait changes observed in younger individuals may become more significant with aging due to age-related changes in postural control, leading to balance disturbances.

We also found that HIV+ individuals reported more balance disturbances and were more likely to have polyneuropathy signs and to use specific medications (opiates and sedatives), both of which are strongly associated with balance disturbances. The interaction between HIV serostatus and chronic cannabis use persisted after adjustment for these potential confounding factors. Our findings are consistent with previous research showing that higher rates of falls among HIV+ individuals are related to multiple comorbidities and the use of opiates, sedatives, and antihypertensive medications (Erlandson et al. 2017). Those using cannabis were more likely to use these balance-affecting medications, increasing the risk of drug-drug interactions or synergistic toxicities. A meta-analysis looking at the association between medication classes and fall risk showed the respective odd ratios: 1.24 [1.01–1.50] for antihypertensive agents, 1.47 [1.35–1.62] for sedatives and hypnotics, and 0.96 [0.78–1.18] for narcotics (Article 2009). Participants with history of diabetes, along with a higher prevalence of chronic distal polyneuropathy were more likely to have balance disturbances as well. Diabetes and cDSPN were controlled for in our statistical model and the effect of long-term cannabis use persisted. Therefore, our findings might indicate that HIV+ individuals might be less susceptible to cannabis’ effect on balance. The proportion of participants who ever used cannabis did not differ among HIV+ and HIV− individuals. The proportion of virologic suppression in HIV+ individuals on treatment was low in our population; however, it did not affect the primary outcome (balance disturbance). A positive urine toxicology for THC was not significantly more common in participants with balance disturbances. Urine toxicology is not a good proxy for recency of use or intoxication, since the test may remain positive for up to 30 days.

There are limitations to this study. First, data were based on a retrospectively self-reported data. Second, the cross-sectional design cannot show a causal relationship between cannabis use and balance disturbances. Nevertheless, a recent comparison of fall recall and prospective fall collection among 600 older adults in Germany found nearly identical incidence rate of falls, supporting the validity of retrospective fall history (Rapp et al. 2014). Also, we did not collect any information about cannabis route of administration or whether cannabis use was recreational or prescribed, which factors may moderate cannabis-balance association.

## Conclusions

We found unexpectedly that while long-term cannabis use in HIV− individuals was associated with more severe balance disturbances, there were no associations in HIV+ individuals. This suggests that cannabis use in HIV is safe with respect to balance disturbances. Given that HIV is related to persistent inflammation despite virologic suppression on antiretroviral therapy, future mechanistic studies are needed to determine whether HIV-associated inflammation contributes to the higher prevalence of balance disturbance in HIV+ individuals and whether cannabinoids have anti-inflammatory effects that mitigate HIV-associated balance disturbance.

## Data Availability

The data used to generate our results are available at the HNRP database at the University of California San Diego. The data can be made available by contacting the last author of the manuscript.

## Abbreviations

AIDS: Acquired immunodeficiency syndrome
ART: Antiretroviral therapy
BMI: Body mass index
CBD: Cannabidiol
cDSPN: Chronic distal sensory polyneuropathy
CI: Confidence interval
CIDI: Composite international diagnostic interview
EFV: Efavirenz
HIV: Human immunodeficiency virus
HNRP: HIV Neurobehavioral Research Program
OR: Odds ratio
SD: Standard deviation
TLFB: Timeline follow-back assessment
THC: Tetrahydrocannabinol
